# Energy restriction, exercise and atorvastatin treatment improve endothelial dysfunction and inhibit miRNA-155 in the erectile tissue of the aged rat.

**DOI:** 10.1186/s12986-018-0265-z

**Published:** 2018-04-16

**Authors:** B. Rocha, A. R. Rodrigues, I. Tomada, M. J. Martins, J. T. Guimarães, A. M. Gouveia, H. Almeida, D. Neves

**Affiliations:** 1 Department of Biomedicine - Experimental Biology Unit, Faculty of Medicine, University of Porto, Al. Prof. Hernâni Monteiro, 4200-319 Porto, Portugal; 2 Instituto de Investigação e Inovação em Saúde (I3S) Rua Alfredo Allen, 208, 4200-135 Porto, Portugal; 3 Faculty of Biotechnology, Portuguese Catholic University, Rua Arquiteto Lobão Vital, 4202-401 Porto, Portugal; 4 Hospital CUF Porto, Estrada da Circunvalação, 14341, 4100-180 Porto, Portugal; 5 Department of Biomedicine - Biochemistry Unit, Faculty of Medicine, University of Porto, Al. Prof. Hernâni Monteiro, 4200-319 Porto, Portugal; 6 Institute of Public Health, University of Porto, Rua das Taipas, 135, 4050-600 Porto, Portugal; 7 Clinical Pathology Department of São João Hospital Centre, Porto, Portugal; 8 Faculty of Nutrition and Food Sciences, University of Porto, Rua Dr. Roberto Frias, 4200-465 Porto, Portugal

**Keywords:** Endothelial dysfunction, Energy restriction, Exercise, Atorvastatin, Sirtuins, microRNA-155

## Abstract

**Background:**

Endothelial dysfunction underlies cardiovascular disease that frequently affects aged individuals. Characterized by local decrease in nitric oxide, it results from down-regulation of endothelial nitric oxide synthase (eNOS) expression/activity. Aiming to elucidate the molecular mechanisms involved in age-related endothelial dysfunction and to unveil potential therapeutic targets, we tested how diet pattern, exercise and atorvastatin modulate the expression of eNOS, inducible NOS (iNOS), endothelin-1, sirtuins (SIRT) and microRNA-155 in the erectile tissue of high-fat fed aged rats.

**Methods:**

Sprague-Dawley male rats fed with high-fat diet until they completed 12 months were grouped and subjected to energy restriction (ER), ER and atorvastatin, or, ER, atorvastatin and physical exercise. Controls were fed with standard rodent chow. The blood pressure was measured using the tail-cuff method before sacrifice at 18 months. Glucose, total cholesterol, HDL, triglyceride and CRP were assessed in blood and eNOS, endothelin-1, iNOS and sirtuins were detected by immunofluorescence in the penis sections; eNOS, endothelin-1, iNOS, SIRT2–4 and SIRT6–7 were semi-quantified by western blotting in tissue homogenates. MicroRNA-155 was quantified using RT-PCR in formalin-fixed paraffin embedded sections. To compare the studied variables, two-tail student *t* test was used.

**Results:**

Atorvastatin promotes eNOS expression and is more efficient than ER or exercise in the control of hyperlipidemia and inflammation. Among the studied sirtuins, detected for the first time in the erectile tissue of the aged rat, SIRT2 aligns with eNOS expression. Both proteins exhibit over-expression in animals with combined exercise, atorvastatin and ER. Analysis of microRNA-155 expression also suggests its intervention in the regulation of eNOS expression. ER, particularly when combined with atorvastatin, was able to reverse the increase of iNOS and endothelin-1 in high-fat fed rats.

**Conclusions:**

The present results indicate that the association of ER, atorvastatin and exercise is more efficient than isolated interventions in the prevention of endothelial dysfunction.

**Electronic supplementary material:**

The online version of this article (10.1186/s12986-018-0265-z) contains supplementary material, which is available to authorized users.

## Background

Ageing and obesity increase susceptibility to atherosclerosis and cardiovascular diseases (CVD) [1]. The asymptomatic antecedent of these entities that insidiously develop along time, is endothelial dysfunction (ED) [2], which is characterized by a decrease in bioavailability of nitric oxide (NO). NO is a gaseous molecule, constitutively produced by endothelial NO synthase (eNOS) in endothelial cells, that diffuses into the smooth muscle cell (SMC) layer and induces its relaxation [3]. Under pro-inflammatory conditions, inducible NOS (iNOS) is activated, forming high levels of NO and exhausting the substrate and co-factor shared with eNOS. Additionally, high levels of iNOS-derived NO interact with reactive oxygen species (ROS) and cause oxidative damage. Opposite to NO, endothelin-1 (ET-1) is a powerful endothelium-derived vasoconstrictor that contributes to ED and oxidative stress [4].

Decrease in eNOS-derived NO relates to functional modifications that occur with variable extension in different vascular districts. The small vessels of the *corpus cavernosum* (CC) of the penis are particularly susceptible to endothelial structural/functional integrity loss [5, 6]. Hence, the CC constitutes a valuable feature to study age- and diet-related vascular modifications [7, 8].

Several strategies may be considered to prevent age-related ED, including diet modification, exercise or pharmacological interventions. Energy restriction (ER), i.e. reduced energy intake without malnutrition, counteracts most age-related modifications in cells and individuals, preserving endothelium-dependent dilation in rodents through maintaining NO bioavailability [9]. Physical exercise also has favorable effects on endothelial function given its potent anti-inflammatory [10] and eNOS upregulatory functions [2].

Hydroxymethylglutaryl-coenzyme A reductase inhibitors (statins), besides cholesterolemia-lowering effects, seem to improve endothelial function. The exact mechanism is unclear but it seems to mitigate oxidative stress conditions in vessel walls [11]. Statins stabilize eNOS mRNA in endothelial cells, up-regulate eNOS activity through PI3K-Akt-mediated Serine^1177^ phosphorylation [12], reduce ET-1 production, exert anti-inflammatory effects downregulating iNOS activation [13] and thus ameliorate endothelial function [14].

Besides PI3K-Akt, other endogenous factors have been recognized as modulators of eNOS activity. Sirtuin1, a NAD^+^-dependent histone deacetylase, member of the mammalian sirtuin family (SIRT1–7) activates eNOS through deacetylation [15]. Recent evidence suggest that sirtuins may act together to correct spontaneous acyl modifications that occur under metabolic stress conditions [16].

MicroRNAs (miRNAs) regulate gene expression by binding preferentially to 3′-untranslated regions of multiple targets. Nevertheless, miRNAs seem to have a tissue- and cell-specific expression pattern. MiRNA-155 (miR-155) is constitutively expressed in endothelial cells and extremely up-regulated in atherosclerotic plaques [17]. In silico analysis suggest that eNOS mRNA may be a direct target of miR-155. Moreover, simvastatin prevented a decrease in eNOS expression, while decreasing miR-155 levels, suggesting miR-155 intervention in the simvastatin-induced increase of eNOS expression [18].

This study aimed to assess: a) SIRT1–7 and miR-155 expression in the CC of the aged rat; b) how diet pattern, exercise and atorvastatin modulate SIRT1–7 and miR-155 expression levels and; c) how miR-155 correlates with the eNOS expression levels in the erectile tissue.

## Methods

### Experimental groups

Twenty-five male Sprague-Dawley rats weighing 200-250 g (Charles River, Barcelona, Spain) were housed individually and kept under a controlled standard environment (12/12 h light/dark cycle; 20–22 °C temperature; 40–60% humidity) with free access to tap water throughout the experiments.

At 2 months of age, the animals were divided into experimental groups according to the flowchart. The flowchart does not correspond to the previously submitted. The upper arrows are not in the correct place.

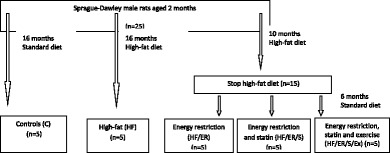



Control group rats (C; *n* = 5) had free access to a standard rodent chow with 4% of energy provided by fat, mostly derived from fish (A04 Panlab®SL, Barcelona, Spain) whereas high-fat diet (HF; *n* = 5) group had free access to a purified rodent diet with 45% of energy from fat derived from lard (58 V8 Test Diet®, Purina Mills®, LLC, PMI Nutrition International®, Richmond, USA) during 16 months.

The remaining 15 rats were maintained with free access to HF diet during ten months and then were randomly divided in three groups (*n* = 5). One group of rats was subjected to a six-month period of energy restriction (ER), consisting of 75% of the daily amount of standard rodent chow consumed by the controls, individually adjusted to body weight (HF/ER; *n* = 5); another group was submitted to ER coupled with treatment with atorvastatin (Pfizer, New York, USA), orally administrated (5 mg/Kg of body weight/day), a dose demonstrated to be sufficient to mitigate dyslipidemia and safe to use in the long-term [19] (HF/ER/S; n = 5); and the third group was subjected to ER coupled with the atorvastatin treatment and a 1 h swim 3 times per week [20] (HF/ER/S/Ex; n = 5).

By the 18th month, all animals were weighed and sacrificed by decapitation after a 16 h fasting. The trunk blood was collected, centrifuged (1000×g for 30 min at 4 °C) and the plasma was recovered and stored at − 80 °C for biochemical determinations. The penises were excised and divided into two fragments: one was immediately stored at − 80 °C for molecular analysis and the other was fixed in 10% buffered formaldehyde for immunofluorescence studies.

### Blood pressure assessment

Systolic (SBP) and diastolic blood pressure (DBP) were measured using the tail-cuff method in conscious rats (LE5008-05PL, Panlab S.I., Barcelona, Spain) when they completed 18 months of age. Measurements were made 10–15 min after acclimatization under restraining conditions and were repeated in three consecutive days. Data from the third day were considered valid.

### Biochemical determinations

Plasma glucose concentrations were determined using a glucose analyzer (OneTouch® Ultra™, Lifescan, Inc., Milpitas, CA, USA) at the experimental end-point. Plasma total cholesterol (TC), high-density lipoprotein cholesterol (HDL-c) and triglyceride (TG) concentrations were determined by enzymatic colorimetry in an Olympus® auto-analyzer (Olympus America, Inc., NY, USA) resorting to commercial kits (OSR6516, OSR6587, and OSR61118, Olympus America, Inc., for TC, HDL-c and TG, respectively). Low-density lipoprotein cholesterol (LDL-c) levels were calculated by the formula of Friedewald (LDL-c = TC − [HDL-c − 1/5 TG], if serum TG is 400 mgdL^− 1^ or less) [21].

C-reactive protein (CRP) levels in serum were analyzed with an immunoturbidimetric latex CRP assay, normal set (OSR6199, Olympus America, Inc.).

### Immunofluorescence

Briefly, after fixation for 24 h, the penis fragments were dehydrated in a series of aqueous ethanol solutions with increasing concentration, embedded in paraffin and oriented along their transversal axis. Sections with thickness of 4-6 μm were cut in a microtome (RM 2145, Leica Microsystems GmbH, Germany) and placed on 0.1% poly-L-lysine-coated microscopy slides. For immunofluorescence (IF) detection of α-actin (a SMC marker), eNOS, ET-1, iNOS, SIRT1, SIRT2, SIRT3, SIRT4, SIRT5, SIRT6, and SIRT7, the penis sections were deparaffinized in xylene, rehydrated in aqueous ethanol solutions (v/v) with decreasing concentration (100%, 90% and 70%), exposed to 1 M HCl solution for epitope retrieval, neutralized with 0.1 M borax solution, followed by 1 h incubation with blocking solution (1% w/v bovine serum albumin (BSA) in phosphate-buffered saline, PBS). Afterwards, sections were incubated overnight at 4 °C with a mixture of primary antibodies: rabbit anti-iNOS (Abcam, Cambridge, UK) with goat anti-ET-1 (Santa Cruz Biotechnology, Inc., CA, USA); rabbit anti-eNOS (Santa Cruz Biotechnology, Inc) with mouse anti-α-actin (Millipore, Darmstadt, Germany); rabbit anti-SIRT1 (Santa Cruz Biotechnology, Inc.) with goat anti-SIRT7 (Santa Cruz Biotechnology, Inc.); and mouse anti-α-actin with rabbit anti-SIRT2 (Sigma-Aldrich Co, Dorset, UK), anti-SIRT3 (Cell Signaling Technology, MA, USA), anti-SIRT4 (Sigma-Aldrich Co.), anti-SIRT5 (Sigma-Aldrich Co.) or anti-SIRT6 (Sigma-Aldrich Co.) Negative controls were performed without primary antibodies. After washing with PBS, the tissue sections were incubated for 1 h at room temperature in a humidity chamber, with a suitable mix of secondary antibodies diluted 0.05% (v/v), either anti-rabbit conjugated with Alexa Fluor A488® (green) and anti-goat conjugated with Alexa Fluor A568® (red) or anti-mouse conjugated with Alexa Fluor A568® (red) (Molecular Probes, Leiden, Netherlands). Nuclei were counterstained with 4′,6-diamidino-2-phenylindole (DAPI; blue) (Molecular Probes). Tissue sections were then mounted in a buffered solution of glycerol and observed in an ApoTome fluorescence microscope (Imager. Z1, Carl Zeiss MicroImaging GmbH, Göttingen, Germany). IF analysis was carried out in 3 different animals of each experimental group and representative images of each group were selected. All images were acquired with AxionVision® software (Carl Zeiss MicroImaging, GmbH).

### Western blotting

For protein analysis, the penis fragments were mechanically homogenized in lysis buffer (0.1 M NaCl, 5 mM EDTA and 0.5% (v/v) Triton X-100 in 50 mM Tris pH 7.2) supplemented with 0.5% (v/v) protease inhibitor cocktail (P8340, Sigma-Aldrich Co.) using a Polytron (Polytron Pt 2500E, Kinematica, Switzerland) until the residual fragments of the tissue had lost their reddish color. After sonication for 15 min (Bioruptor™ UCD-200, Diagenode, Belgium) and centrifugation at 12000 x g for 30 min, total protein quantification was performed for each sample as described by Bradford (1976) [22] using Bradford reagent (BioRad Laboratories, Hercules, CA, USA). Absorbance was read at 595 nm in a Tecan Infinite® 200 PRO microplate reader (Tecan, Männedorf, Switzerland). Then, a total of 20 μg of protein from each sample diluted in loading buffer was heated at 65 °C for 30 min in a thermal shaker adjusted to 400 rpm (Thermomixer® 5436, Eppendorf Ibérica, Madrid, Spain), followed by 5 min at 95 °C, centrifuged at 15000×g for 5 min (Eppendorf Ibérica), and then separated by sodium dodecylsulfate-polyacrilamide gel electrophoresis (SDS-PAGE), using the discontinuous buffer system described by Laemmli (1970) [23] and a 12% acrylamide resolving gel (BioRad Laboratories) for approximately 1 h at room temperature under constant current of 25 mA. After electrophoresis, peptides were electrically transferred to a nitrocellulose membrane with a pore size of 0.45 μm (Trans-Blot® Transfer Medium BioRad Laboratories) in a BioRad system, for 1 h and 30 min at constant voltage (30 V). The peptides in the nitrocellulose membrane were further reversibly stained with Ponceau S solution and the image of the membrane was captured in a ChemiDoc TM XRS (BioRad Laboratories). The membrane was then washed, incubated first with blocking solution (5% (w/v) of bovine serum albumin in Tris buffer saline) and then with the aforementioned primary antibodies diluted in blocking solution for at least 48 h at 4 °C with constant agitation. After washing, the membranes were incubated with the proper secondary antibodies coupled with Horseradish Peroxidase (HRP) diluted in blocking solution for 1 h at room temperature with constant agitation. Next, the nitrocellulose membranes were washed and the signal detection was performed using a chemiluminescent peroxidase substrate (Clarity™ Western ECL substrate, BioRad Laboratories). Protein bands were visualized and the images captured in a ChemiDocTM XRS apparatus (BioRad Laboratories). Intensity of bands was quantified by densitometry using the Image Lab® software (BioRad Laboratories). Protein expression levels were normalized to the total protein stain using Ponceau S in the respective lane. Each experiment was repeated four times.

### MicroRNA quantification by real-time polymerase chain reaction

Total RNA extraction was performed using the commercial Recover All™ Total Nucleic Acid Isolation Kit (Ambion, Austin, Texas), according to the instructions of the manufacturer with slight modifications, as proposed by Liu and Xu [24]. In brief, for each sample, five formalin-fixed paraffin embedded tissue sections (15 μm thick) were cut in a microtome, using a fresh microtome blade for each sample. Excess paraffin was trimmed and each sample was deparaffinized in 100% xylene, washed with 100% ethanol, and incubated with a protease for 2 h at 50 °C, followed by 15 min at 75 °C to allow sample mixtures to clarify. After DNase digestion, 7 μL of total RNA were used to generate cDNA using the MysticCq microRNA cDNA synthesis Mix (Sigma-Aldrich Co.). In the process of cDNA synthesis by reverse transcriptase (RT) reaction, miRNAs were initially subjected to polyadenylation by poly (A) polymerase that catalyzed the transfer of adenosine deoxynucleotides to the 3′-end of all RNAs, including miRNAs. Then, the RNAs were converted into cDNA by RT using an oligo-dT adaptor primer, incorporating a unique sequence at its 5′-end that would later be recognized by a Universal primer, which allowed for the amplification of cDNAs in real-time PCR reactions. Each qPCR reaction mix consisted of 1 μL of RT product, 6 μL SYBR Green (SYBR® Select Master Mix, Applied Biosystems, CA, USA), 0.6 μL of lower Universal primer, 1 μL of the upper primer for miR-155 (miScript Primer Assays for miRNA-155-5p, Qiagen, Germany) and 3.4 μL of water. Reactions were carried out in 96-well thin-wall PCR plates in the StepOnePlus™ Real-time PCR system (Life Technologies, CA, USA). The amplification conditions were as follows: 95 °C for 10 min; 95 °C for 15 s, 55 °C for 30s and 60 °C for 30s, for 42 cycles. All samples were run in duplicate. The cDNA amount for each reaction was normalized with the internal control RNU1A, using the RNU1A primer assay (Qiagen, Hilden, Germany). An amplification reaction control without the RT enzyme (referred to as -RT) was performed in order to discard a possible genomic DNA contamination in the RNA solution. Relative gene expression was calculated from the formula 2^ΔCT^ (ΔCT = CT_RNU1A_ - CT_target_).

### Statistical analysis

Results are presented as a mean value ± standard error (SEM). To compare the studied variables, two-tail student *t* test was used, considering *p* ≤ 0.05 as statistically significant for every comparison.

## Results

### Body weight and blood pressure evaluation

Data relative to body weight and blood pressure of groups C, HF and HF/ER were previously published [7]. For a better interpretation of the results they were included in Table 1. No differences were observed among C, HF and HF/ER groups regarding body weight. However, treatment with ER and atorvastatin (HF/ER/S) resulted in a reduction of the body weight of rats at the experimental endpoint, compared to both HF rats (*p* = 0.037), HF/ER (*p* = 0.005) and also controls (p = 0.005). Exercise does not appear to have additional effects relatively to ER associated with atorvastatin. Regarding systolic and diastolic blood pressure levels, group HF/ER/S/Ex presented lower levels of systolic blood pressure than HF/ER/S (p = 0.037), HF/ER (*p* = 0.009) and HF (*p* = 0.001) groups. A decrease was found in the diastolic blood pressure level in the HF/ER/S group when compared to HF-treated rats (*p* = 0.003).

**Table 1:** Body weight, blood pressure and biochemical determinations in blood

	C	HF	HF/ER	HF/ER/S	HF/ER/S/Ex
Final body weight (g)	748.6 ± 33.8	888.8 ± 91.4	681.6 ± 16.1	584.4 ± 8.3^*,#,+^	605.6 ± 15.2^*,+^
Systolic blood pressure (mmHg)	104.2 ± 2.0	128.6 ± 1.5	133.5 ± 6.3	130.3 ± 7.2^*^	104.6 ± 1.3^#,+,$^
Diastolic blood pressure (mmHg)	90.8 ± 3.4	103.6 ± 2.2	83.5 ± 5.6	79.3 ± 2.3^#^	82.8 ± 8.2
Glycemia (mg/dL)	105.2 ± 12.4	135.8 ± 5.4	111.0 ± 5.7^#^	129.5 ± 5.7	94.0 ± 2.8^#^
Total cholesterol (mg/dL)	104.0 ± 13.6	167.4 ± 20.3^*^	161.5 ± 12.9^*^	124.5 ± 10.4^+^	88.2 ± 9.5^#,+^
HDL-c (mg/dL)	62.4 ± 7.5	85.4 ± 8.6	82.8 ± 4.3^*^	46.0 ± 10.8^#,+^	46.8 ± 5.4^#,+^
LDL-c (mg/dL)	28.0 ± 11.0	40.6 ± 8.3	51.8 ± 10.7	33.3 ± 5.5	38.6 ± 3.1
Triglycerides (mg/dL)	155.3 ± 27.3	207.2 ± 40.8	150.0 ± 16.4	106.8 ± 4.6^*,#^	71.0 ± 5.2^#,+,$^
CRP (mg/L)	3.6 ± 0.5	8.4 ± 1.0^*^	3.6 ± 1.1^#^	1.8 ± 0.2^*,#,+^	2.4 ± 0.1^*,#^

Data are presented as mean ± SEM. Data relative to groups C, HF and HF/ER were previously published [7]

*C*: controls; *HF*: high-fat; *HF/ER*: high fat/energy restriction; *HF/ER/S*: high-fat/energy restriction/statin;: high-fat/energy restriction/statin/exercise

^*^p < 0.05 vs C, ^#^ *p* < 0.05 vs HF, ^+^ *p* < 0.05 vs HF/ER, ^$^*p* < 0.05 vs HF/ER/S

### Biochemical analysis

Blood levels of glucose, TC, HDL-c, LDL-c, TG and CRP, of groups C, HF and HF/ER were previously published [7] (Table1). Regarding glycemia, HF/ER and HF/ER/S/Ex groups presents a decrease relatively to the HF-treated animals (*p* = 0.010 and *p* = 0.011, respectively). No other differences were verified among experimental groups.

Broadly, our data indicate that atorvastatin treatment was efficient in lowering blood TC, HDL-c and TG. HF/ER/S and HF/ER/S/Ex groups presented TC levels lower than those observed in HF/ER rats (*p* = 0.048 and *p* = 0.027, respectively). TC levels in HF/ER/S/Ex animals were lower when compared to the HF group (p = 0.048). No differences were observed among groups for LDL-c levels in blood, but a decrease in HDL-c levels in atorvastatin-treated rats was found relatively to those in the HF diet or HF/ER groups (*p* = 0.040 and *p* = 0.036 versus HF, and *p* = 0.043 and *p* = 0.013 versus HF/ER, for HF/ER/S and HF/ER/S/Ex, respectively). Similarly, a decrease in TG levels was observed in HF/ER/S when compared to those treated with HF diet (*p* = 0.031) or controls (*p* = 0.007). Exercise exerted an additional lowering effect on TG relatively to atorvastatin associated with ER (*p* = 0.002).

CRP is an important marker of the inflammatory state. Both HF/ER/S and HF/ER/S/Ex groups presented lower levels of CRP than HF-treated rats (*p* = 0.005 and *p* < 0.001, respectively) and controls (*p* = 0.047 and *p* = 0.010, respectively). HF/ER/S also presented lower levels than the HF/ER group (*p* = 0.020).

### Dual immunolabeling of eNOS/α-actin; iNOS/ET-1; SIRT1/SIRT7; SIRT2/α-actin; SIRT3/α-actin; SIRT4/ α-actin; SIRT5/ α-actin and SIRT6/ α-actin

**Fig. 1:**
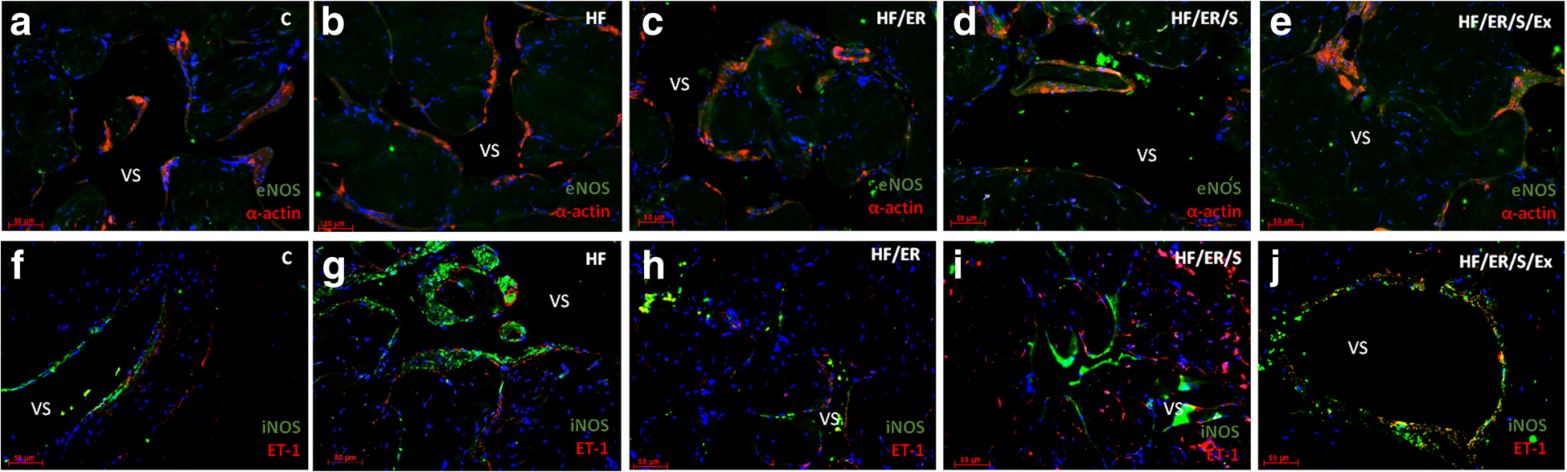
Dual immunolabeling of eNOS/α-actin (**a-e**) and iNOS/ET1 (**f-j**) in erectile tissue of rats from all experimental groups (*n* = 3/group). Smooth muscle cells (SMC) layer was evident after α-actin labeling (red), surrounding endothelium (not labeled) and vascular spaces in all animals. No marked differences among groups were evident. eNOS was detected not only in the endothelium, but also in SMC (green) apparently with higher intensity in HF/ER, HF/ER/S and HF/ER/S/Ex groups (**c-e**). iNOS was detected in the SMC (green) in all experimental groups (**f-j**), being particularly evident in HF-treated rats (**g**). ET-1 was mostly localized in the endothelium (red), with an apparent higher intensity in HF-fed rats (**g**). C-control; HF-high-fat diet treated rats; HF/ER-high-fat diet treated rats under energy restriction for 6 months; HF/ER/S-high-fat diet treated rats under energy restriction and atorvastatin treatment for 6 months; HF/ER/S/Ex-high-fat diet treated rats under energy restriction, atorvastatin treatment and exercise for 6 months. VS- vascular space

**Fig. 2:**
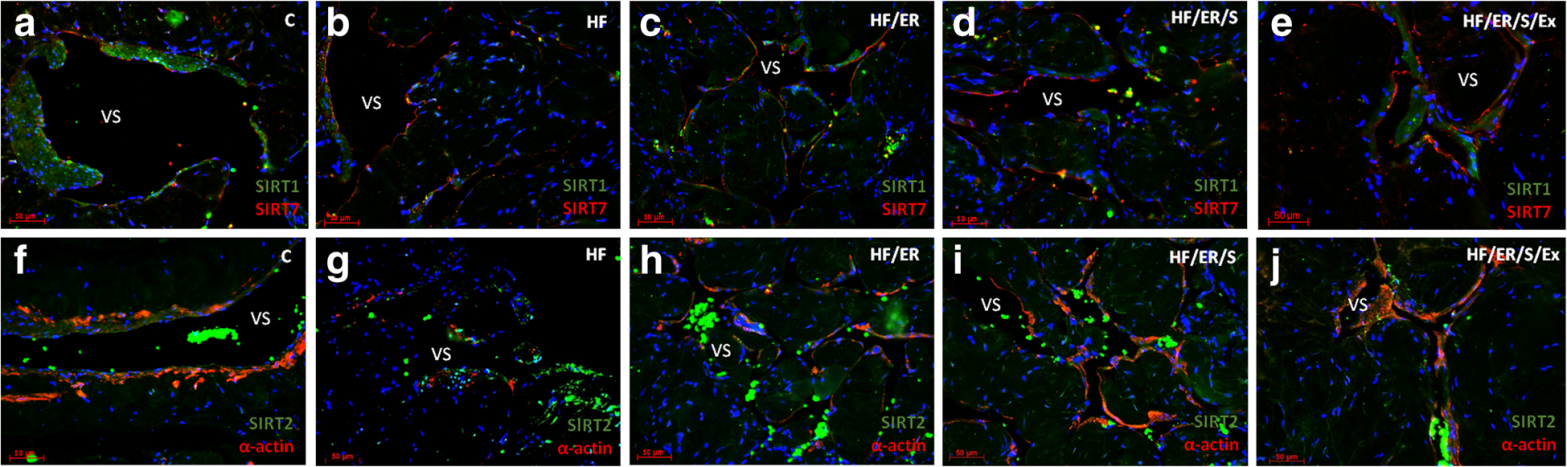
Dual immunolabeling of SIRT1/SIRT7 (**a-e**) and SIRT2/α-actin (**f-j**) in erectile tissue of rats from all experimental groups (*n* = 3/group). SIRT1 was identified in the nucleus and cytoplasm (green) mainly in the SMC of all the analyzed tissues (**a-e**) and SIRT7 is apparently more expressed in the endothelium (red) (**a-e**). No marked differences were seen among groups for SIRT1 and SIRT7 expression. SIRT2 expression was observed in cytosol mainly in SMC in all experimental groups, often co-localizing with α-actin labeling (**f-j**). The co-localization seems to be more intense in CC from rats of HF/ER/S/Ex group (**j**). C-control; HF-high-fat diet treated rats; HF/ER-high-fat diet treated rats under energy restriction for 6 months; HF/ER/S-high-fat diet treated rats under energy restriction and atorvastatin treatment for 6 months; HF/ER/S/Ex-high-fat diet treated rats under energy restriction, atorvastatin treatment and exercise for 6 months. VS- vascular space

**Fig. 3:**
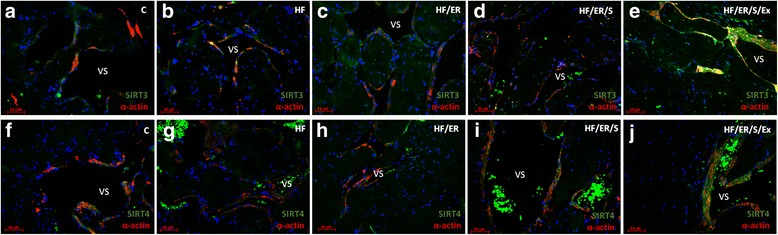
Dual immunolabeling of SIRT3/α-actin (**a-e**) and SIRT4/α-actin (**f-j**) in erectile tissue of rats from experimental groups (*n* = 3/group). Sirtuins were labeled in green and α-actin in red. Mitochondrial SIRT3 and 4 were detected in co-localization with α-actin in the CC of all experimental groups (**a-j**). C-control; HF-high-fat diet treated rats; HF/ER-high-fat diet treated rats under energy restriction for 6 months; HF/ER/S-high-fat diet treated rats under energy restriction and atorvastatin treatment for 6 months; HF/ER/S/Ex-high-fat diet treated rats under energy restriction, atorvastatin treatment and exercise for 6 months. VS- vascular space

**Fig. 4:**
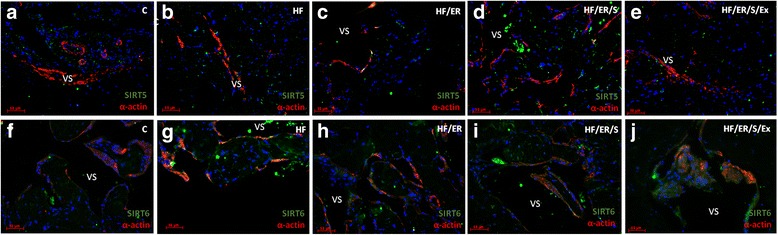
Dual immunolabeling of SIRT5/α-actin (**a-e**) and SIRT6/α-actin (**f-j**) in erectile tissue of rats from experimental groups (n = 3/group). Sirtuins were labeled in green and α-actin in red. Mitochondrial SIRT 5 was detected in the CC of all experimental groups with low co-localization with α-actin (**a-e**). SIRT6, apart from its nuclear labeling, presents a diffuse cytoplasmic labeling in all experimental groups (**f-j**). C-control; HF-high-fat diet treated rats; HF/ER-high-fat diet treated rats under energy restriction for 6 months; HF/ER/S-high-fat diet treated rats under energy restriction and atorvastatin treatment for 6 months; HF/ER/S/Ex-high-fat diet treated rats under energy restriction, atorvastatin treatment and exercise for 6 months. VS- vascular space

Immunolabeling of α-actin confirmed the distribution of SMC in the CC of the rat. The smooth muscle layer was evident in animals from all experimental groups, surrounding the endothelium (not labeled) and vascular spaces. No marked differences among groups were evident (Fig.1a-e, Fig. 2f-j, Fig. 3a-j and Fig. 4a-j). Regarding eNOS, this enzyme was detected in green not only in the endothelium, but also in SMC, in line with previous findings [8], apparently with higher intensity in HF/ER, HF/ER/S and HF/ER/S/Ex rats (Figs. 1c-e). iNOS was detected in the SMC in all the experimental groups (Figs. 1f-j), being particularly evident in HF-treated rats (Fig. 1g). On the other hand, ET-1 was mostly localized in the endothelium, with an apparent higher intensity in HF-fed animals (Fig. 1g). Sirtuins 1–7 were detected in the erectile tissue of the rat in all the experimental conditions (Figs. 2a-j, Figs. 3a-j and Figs. 4a-j). Except for SIRT1, which has previously been identified [8], to the best of our knowledge, the other isoenzymes were demonstrated for the first time in the erectile tissue of the rat in this study. SIRT1 was identified in the nucleus and cytoplasm mainly in the SMC of all the analyzed tissues (Figs. 2a-e), which agrees with our previous findings that demonstrated the co-localization of SIRT1 and α-actin [8]. SIRT7 was apparently more expressed in the endothelium, presenting a distribution compatible with the expected nucleolar localization (Figs. 2a-e). However, cytoplasmic labeling for SIRT7 was also evident. This pattern of distribution was previously detected in the CC of human origin despite the higher expression of SIRT7 observed in the SMC of human [25]. No marked differences were seen among groups for SIRT1 and SIRT7 expression. SIRT2 expression was observed in the cytosol mainly in SMC in all experimental groups, often co-localizing with α-actin labeling (Figs. 2f-j). This co-localization seemed to be more intense in the CC from rats of the HF/ER/S/Ex group (Fig. 2j). Mitochondrial SIRT3, 4 and 5 were detected in the CC of all experimental groups (Figs. 3a-j and Figs 4a-e). The punctuated labeling, previously observed in the CC of human [25], was better seen in SMC. Co-localization of SIRT3 or 4 and α-actin was more evident when compared to SIRT5 and α-actin, possibly because of the low levels of SIRT5 fluorescence (Figs. 4a-e). Regarding SIRT6, apart from its nuclear labeling, a diffuse cytoplasmic labeling was observed in the CC from rats from all groups (Figs 4f-j).

### Semi-quantification of eNOS, iNOS, ET-1, SIRT2, SIRT3, SIRT4, SIRT6 and SIRT7 by western blotting

**Fig. 5:**
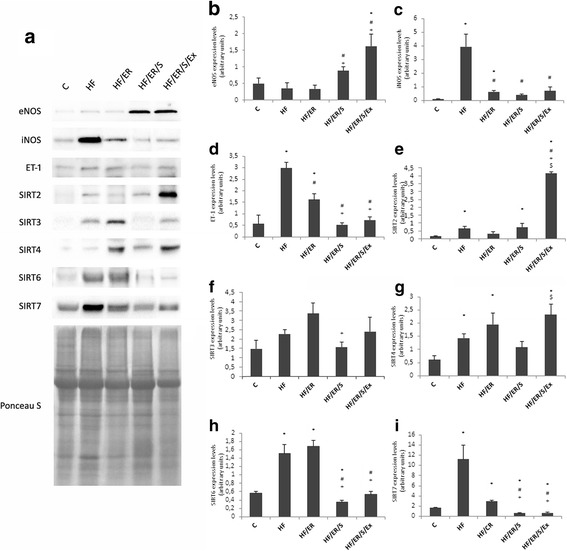
Semiquantification of protein expression levels of the eNOS, iNOS, ET-1, and SIRT2,3,4,6 and 7 by western blotting. Representative blots for each studied protein and representative Ponceau S staining for samples of each group are ilustrated (**a**). Graphs (**b-i**) represent the densitometric quantification of each band relatively to the respective lane after Ponceau S staining. C-control; HF-high-fat diet treated rats; HF/ER-high-fat diet treated rats under energy restriction for 6 months; HF/ER/S-high-fat diet treated rats under energy restriction and atorvastatin treatment for 6 months; HF/ER/S/Ex-high-fat diet treated rats under energy restriction, atorvastatin treatment and exercise for 6 months. Error bars represent standard error for the mean (*n* = 5/group). ^*^ *p* < 0.05 vs C, ^#^ *p* < 0.05 vs HF, ^+^ *p* < 0.05 vs HF/ER, ^$^*p* < 0.05 vs HF/ER/S

Semi-quantification of the expression levels of the studied proteins was carried out by western blotting (Fig. 5). For all the analyzed proteins, a band with the expected molecular weight was identified (Fig. 5a). We verified that the treatment for 6 months with atorvastatin alone or coupled with exercise in rats under ER regimen, after exclusive consumption of HF diet during 10 months, (HF/ER/S and HF/ER/S/Ex groups, respectively) resulted in an increment of eNOS expression, versus HF/ER (*p* = 0.016 and *p* = 0.006, respectively) or HF (*p* = 0.045 and *p* = 0.017, respectively) groups (Fig. 5b). iNOS expression strongly increased in rats under HF diet consumption relatively to the controls (*p* = 0.015). However, the ER regimen associated or not with atorvastatin or exercise (groups HF/ER, HF/ER/S and HF/ER/S/Ex) resulted in a decrease of iNOS expression relatively to the HF group (*p* = 0.003, *p* = 0.002 and *p* = 0.006, respectively) (Fig. 5c). The differences in ET-1 expression among experimental groups varied in an equivalent fashion compared to those observed for iNOS, with an increase in HF relatively to controls (*p* = 0.004), and a decrease in HF/ER, HF/ER/S and HF/ER/S/Ex groups relatively to the HF-fed rats (*p* = 0.013, *p* < 0.001 and *p* < 0.001, respectively) (Fig. 5d).

Sirtuins expression was also analyzed. SIRT1 expression in the rat CC was previously reported [8]. No differences in groups treated with ER combined with atorvastatin or exercise relatively to HF fed rats were found (Additional file 1). SIRT2 expression levels increased in the CC of HF rats relatively to controls (*p* = 0.011) (Fig. 5e). Exercise apparently induced a strong effect in SIRT2 expression, taking into account that HF/ER/S/Ex presented the highest levels (*p* < 0.001, relatively to HF, HF/ER and HF/ER/S) (Fig. 5e). SIRT3 expression decreased in the CC of rats treated with ER and atorvastatin (group HF/ER/S), relatively to those under ER (HF/ER) (*p* = 0.020) (Fig. 5f). HF diet increased SIRT4 expression when compared to controls (*p* = 0.008) and none of the interventions used in this study reversed this effect (Fig. 5g). By contrast, an increment in SIRT4 levels was seen in HF/ER/S/Ex, compared to HF/ER/S rats (*p* = 0.023). SIRT6 expression increased in HF-treated animals compared to controls (*p* = 0.005) (Fig. 5h), an effect that was reversed by atorvastatin treatment (*p* = 0.002 for HF versus HF/ER/S/Ex, and *p* < 0.001 for the other comparisons) (Fig. 5h). SIRT7 variation level was similar to SIRT6 (Fig. 5i), increasing in HF when compared to controls (*p* = 0.04) and decreasing in both groups of atorvastatin-treated rats (HF/ER/S and HF/ER/S/Ex) compared to HF (*p* = 0.011 and *p* = 0.023, respectively) or HF/ER animals (*p* < 0.001 for both) (Fig. 5i).

### MiRNA-155 quantification by real-time polymerase chain reaction

**Fig. 6:**
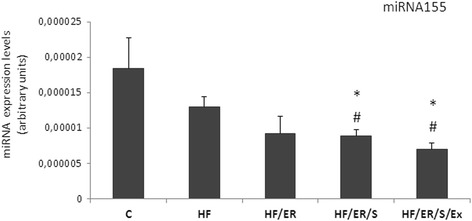
Quantification of miR-155 in the CC of rats from all experimental groups. Relative gene expression was calculated from the formula 2^ΔCT^ (ΔCT = CT_RNU1A_ - CT_target_).C-control; HF-high-fat diet treated rats; HF/ER-high-fat diet treated rats under energy restriction for 6 months; HF/ER/S-high-fat diet treated rats under energy restriction and atorvastatin treatment for 6 months; HF/ER/S/Ex-high-fat diet treated rats under energy restriction, atorvastatin treatment and exercise for 6 months. Error bars represent standard error for the mean (*n* = 4/group). ^*^ *p* < 0.05 vs C, ^#^ *p* < 0.05 vs HF

On account of its apparent intervention in the regulation of eNOS expression, miR-155 was quantified in the CC of rats from all the experimental groups (Fig. 6). We found no differences in miR-155 expression between rats under HF diet and controls, but treatments with atorvastatin or atorvastatin coupled with exercise decreased the expression of miR-155 when compared to HF (*p* = 0.049 and *p* = 0.011, for HF/ER/S and HF/ER/S/Ex, respectively). No differences among HF/ER, HF/ER/S and HF/ER/S/Ex were found.

## Discussion

In previous studies, we demonstrated that long-term HF consumption induces metabolic syndrome in aged rats and that ER could be a non-pharmacological strategy to mitigate most of HF-induced metabolic features [7]. In addition, we found that HF significantly decreases eNOS phosphorylation at Serine^1177^, likely compensated by an upregulation of phosphorylation at Serine^615^, but without NO production increment. An increase in systemic inflammatory markers and upregulation of iNOS was also observed [7, 8].

In the present study we demonstrated that additional strategies, such as atorvastatin treatment or exercise practice, coupled with ER are able to reduce body weight relatively to controls, HF-treated animals and rats that underwent ER for 6 months. As expected, association of atorvastatin and exercise to ER, significantly diminished the levels of circulating TC, HDL-c and TG. In fact, while ER alone did not affect these parameters comparatively to rats exclusively fed with HF diet [7], its association to atorvastatin treatment decreased TG, in comparison to controls and HF rats, HDL-c comparatively to HF and HF/ER rats, and TC relatively to HF/ER animals. Exercise promoted TG reduction, in agreement with previous findings in rats under ER and swimming [26]. In addition, atorvastatin ingestion reduced blood CRP levels apart from intensifying the previously observed anti-inflammatory effect of ER [7]. On the other hand, exercise evidenced a predominant effect relatively to statin treatment in lowering glycemia and systolic blood pressure, in line with a recent report [27]. As observed by others, atorvastatin did not affect blood glucose regulation [28]. Taken together, our findings show an overall beneficial effect owing both atorvastatin treatment and exercise practice, when associated to ER in the metabolic profile and inflammatory status of aged rats.

We found that atorvastatin alone or in association with exercise not only induced up-regulation of eNOS, but also led to a decline in the HF-induced upregulation of ET-1. As well, ER alone was able to mitigate the over-expression of both iNOS and ET-1. These findings agree with previous data observed on vascular cultured cells and aortas of aged rats [13, 29]. In line, a recent study in the heart of obese diabetic mice supports the lowering effect of exercise on iNOS expression [30], despite being considered tissue-specific and dependent on the intensity level [31]. Interestingly, exercise alone does not seem to present lowering effects on ET-1, particularly in aged individuals [32]. On the other hand, statins and exercise practice apparently up-regulate eNOS activity [33, 34]. We were not able to confirm eNOS phospho-activation, taking into account that we did not assess neither phospho-eNOS nor NO levels in CC in response to atorvastatin or exercise, which would be of great interest in order to evaluate their impact on endothelial function.

Endothelial NOS plays a major relevance in endothelial function. Besides Akt-mediated phosphorylation, eNOS activity is modulated by SIRT1-catalysed deacetylation [15]. Our data relative to SIRT1 expression levels in CC did not reveal correlations neither to eNOS levels, nor to treatments. This was not an expected finding, considering previous demonstrations of upregulation of SIRT1 expression and activation by ER, statin and exercise [1, 35, 36]. However, partially explaining our results, a study in the aorta of aged rats failed to demonstrate differences in either eNOS or SIRT1 expression with ER, supporting that in aged animals, differences could be more difficult to detect [37].

The inexistence of correlation between eNOS and SIRT1 expression levels, prompted us to evaluate the expression of the other isoenzymes of the mammalian sirtuin family (SIRT2–7). Sirtuins exhibit a specific localization in cells: SIRT1, 6 and 7 are predominantly nuclear, SIRT3, 4 and 5 are mitochondrial and SIRT2 is mainly cytosolic [38]. However, SIRT1, 3 and 7 can be localized in the cytoplasm, SIRT2 and 3 in the nucleus [8, 38–41] and, recently, SIRT5 and 6 were detected in the cytosol [16]. All sirtuins deacetylate substrates in a NAD^+^-dependent fashion [16] and the substrates are often shared among the family members; p53 is a common substrate of both SIRT1 and 7, NF-kB is common to SIRT1 and 6, and H4K16 is substrate of SIRT1, 2 and 3 [38–40, 42–44]. As well, all mitochondrial sirtuins, SIRT3, 4 and 5 intervene in the deacetylation of acyl modifications that occur under metabolic stress, e.g. in nutrient-derived energy imbalance [16]. This hypothesis is based on the evidence that acyl-modifications may occur by non-enzymatic processes in response to high levels of acyl-Coenzyme A in the mitochondrial matrix [45]. Our findings relative to the mitochondrial sirtuins agree with a cooperative activation of transcription of SIRT3 and 4, regarding the equivalent variation of their levels in response to the experimental conditions, despite the absence of significant differences. We found a mild increase of SIRT3 and 4 in HF-treated rats, intensified in rats submitted to ER, their decrease when ER was associated with atorvastatin and an increase following exercise. Unfortunately, we did not observe levels of SIRT5 expression in a sufficient number of animals to carry out statistical analysis.

The cardiovascular protective nuclear SIRT6 and 7 increased substantially in the CC of rats under HF diet, and SIRT7 decreased upon atorvastatin and exercise. An increase in SIRT7 protein, but not SIRT6, in aged individuals with CVD risk factors relatively to young ones has previously been reported [25]. Whilst SIRT6 has been implicated in the delay of ageing phenotype [46], DNA repair [47], and in the prevention of inflammation, ED and cardiac hypertrophy [48], SIRT7 is a major regulator of nuclear-encoded genes involved in mitochondrial function of cardiac cells [48, 49]. In view of the high-increase in SIRT6 and 7 in HF diet-treated rats and their decrements upon atorvastatin and exercise, their activation may indeed constitute a compensatory protective response.

Among the sirtuins, SIRT2 expression demonstrated a variation in response to experimental conditions in the rat CC that partially fits that observed for eNOS. In fact, eNOS expression increases in rats treated with ER coupled to atorvastatin and exercise, compared to HF group. A gradually increased expression in HF/ER/S and HF/ER/S/Ex groups was found for both eNOS and SIRT2, suggesting not only their crosstalk in the CC, but also that exercise and atorvastatin intervene in the regulation of expression of SIRT2. Despite the lack of evidence of a direct effect of SIRT2 on eNOS activation, its role in the control of oxidative stress [50] indicates enhanced NO bioavailability. No previous evidence suggests that atorvastatin or exercise, when individually used, are able to up-regulate SIRT2 expression. An in vitro study demonstrated that rosuvastatin does not promote SIRT2 expression in endothelial cells along 24 h [35]. Also, SIRT2 expression did not increase in the skeletal muscle of female rats subjected to one-year voluntary running intervention [36]. However, no studies have tested the association of statin and exercise effect on SIRT2 expression. Additionally, we should take into consideration that sirtuins present a cell-specific expression and activity and that cells in CC could respond differentially to pharmacological or non-pharmacological interventions relatively to skeletal muscle.

Changes on miR-155 expression in CC nearly opposed the variations found for eNOS, which was expected in view of the predicted affinity of miR-155 to eNOS mRNA [18]. Previous evidence supporting that miR-155 promotes atherosclerosis [17] and decreases endothelium-dependent vasorelaxation through targeting eNOS [18], corroborates our data. A decrease in miR-155 was found in groups treated with ER and atorvastatin associated or not with exercise, but isolated HF diet or 6 months of ER after HF, did not affect eNOS or miR-155 significantly, suggesting that other contributers, besides diet pattern modification are intervening. To the best of our knowledge, no evidence of modulatory effect of atorvastatin, in contrast to simvastatin [18], or exercise on miR-155 has been reported before. As miRNAs are major regulators of gene expression, they are putative targets for cardiovascular diseases prevention or therapy. Recent evidence associates other miRNAs with ED and particularly with the establishment of erectile dysfunction in aged animals and diabetic patients [51–53], which is promissory for the elucidation of the molecular mechanisms associated with this group of diseases.

## Conclusion

Taken together our data demonstrated that among interventions to mitigate ED, atorvastatin is more efficient than ER or exercise in controlling hyperlipidemia and inflammation, as well as promoting eNOS expression in the CC. Conversely, glycemia and blood pressure are predominantly ameliorated by exercise. Additionally, it was found that combined exercise, atorvastatin and ER treatments increment expression of both SIRT2 and eNOS while decrease miR-155 levels in the CC of aged rats.

In conclusion, this study indicates that exercise, atorvastatin and ER in association have potential beneficial effects on preventing ED in HF diet-treated aged rats.

## Additional file

Additional file 1: Semiquantification of SIRT1 expression levels by western blotting. The graph represent the densitometric quantification of SIRT1 band relatively to the respective lane after Ponceau S staining. Representative blots and Ponceau S staining for samples of each group are shown. C-control; HFD-high-fat diet treated rats; HFD/ER-high-fat diet treated rats under energy restriction for 6 months; HFD/ER+S-high-fat diet treated rats under energy restriction and atorvastatin treatment for 6 months; HFD/ER+S+Ex-high-fat diet treated rats under energy restriction, atorvastatin treatment and exercise for 6 months. Error bars represent standard error for the mean (n=5/group). (PNG 135 kb)

## Abbreviations

C: Control
CC: *Corpus cavernosum*
CRP: C-reactive protein
CVD: Cardiovascular diseases
DAPI: 4′,6-diamidino-2-phenylindole
DBP: Diastolic blood pressure
ED: Endothelial dysfunction
eNOS: Nitric oxide synthase
ER: Energy restriction
ET-1: Endothelin-1
Ex: Exercise
HDL-c: High-density lipoprotein cholesterol
HF: High-fat diet
HRP: Horseradish peroxidase
IF: Immunofluorescence
iNOS: inducible NOS
LDL-c: Low-density lipoprotein cholesterol
miRNA: microRNA
NO: Nitric oxide
PBS: Phosphate-buffered saline
ROS: Reactive oxygen species
RT: Reverse transcriptase
RT-PCR: Real-time Polymerase Chain Reaction
S: Atorvastatin
SBP: Systolic blood pressure
SDS-PAGE: Sodium dodecylsulfate-polyacrilamide gel electrophoresis
SEM: Standard error for the mean
SIRT: Sirtuin
SMC: Smooth muscle cell
TC: Total cholesterol
TG: Triglyceride
VS: Vascular space
