# Supplementary figures and images for: Energy restriction, exercise and atorvastatin treatment improve endothelial dysfunction and inhibit miRNA-155 in the erectile tissue of the aged rat
Source: Nutr Metab (Lond). 2018 Apr 16;15:28. doi: 10.1186/s12986-018-0265-z (PMC5902942; doi:10.1186/s12986-018-0265-z)

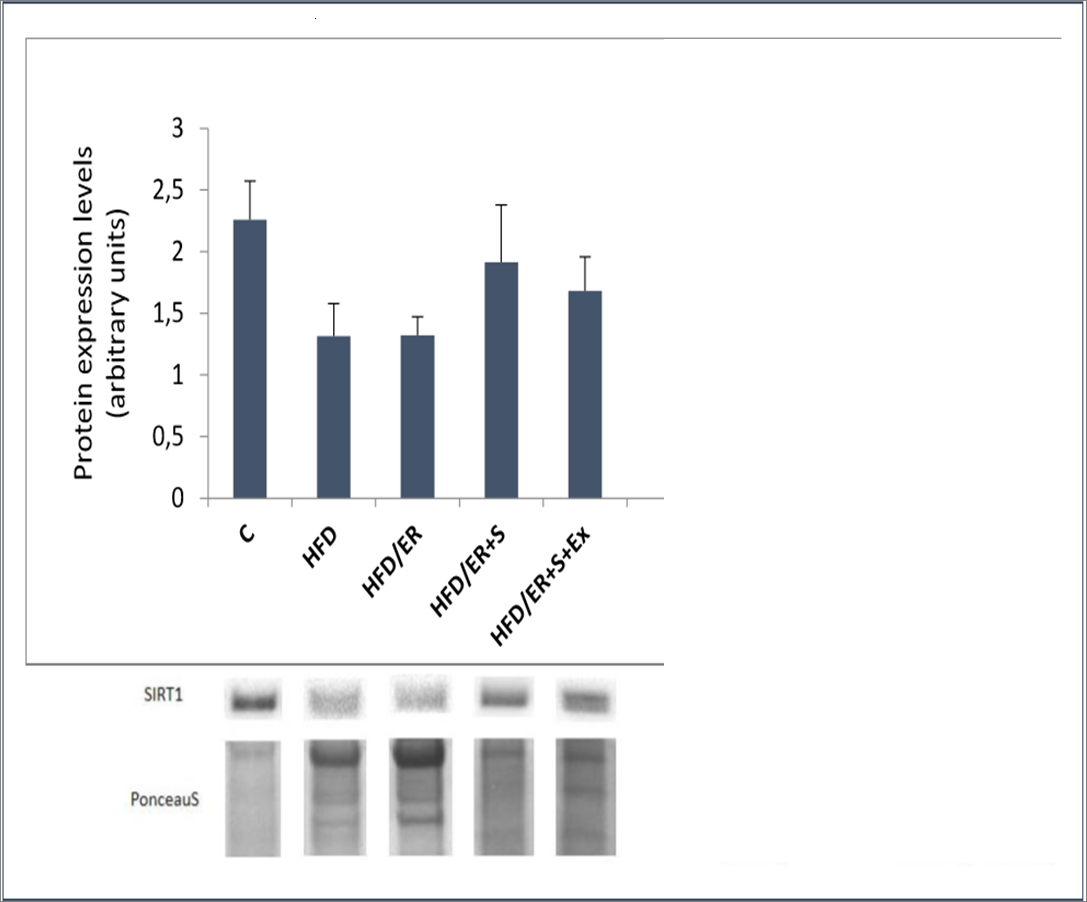

Supplement: Supplementary file 1 — Semiquantification of SIRT1 expression levels by western blotting. The graph represent the densitometric quantification of SIRT1 band relatively to the respective lane after Ponceau S staining. Representative blots and Ponceau S staining for samples of each group are shown. C-control; HFD-high-fat diet treated rats; HFD/ER-high-fat diet treated rats under energy restriction for 6 months; HFD/ER+S-high-fat diet treated rats under energy restriction and atorvastatin treatment for 6 months; HFD/ER+S+Ex-high-fat diet treated rats under energy restriction, atorvastatin treatment and exercise for 6 months. Error bars represent standard error for the mean (n=5/group). (PNG 135 kb) [file 12986_2018_265_MOESM1_ESM.png]
